# Assessing and treating primary headaches and cranio-facial pain in patients undergoing rehabilitation for neurological diseases

**DOI:** 10.1186/s10194-017-0809-z

**Published:** 2017-09-29

**Authors:** Cristina Tassorelli, Marco Tramontano, Mariangela Berlangieri, Vittorio Schweiger, Mariagrazia D’Ippolito, Valerio Palmerini, Sara Bonazza, Riccardo Rosa, Rosanna Cerbo, Maria Gabriella Buzzi

**Affiliations:** 1 IRCCS National Neurological Institute “C. Mondino”, Pavia, Italy; 2 Department of Brain and Behavioural Sciences, University of Pavia, Pavia, Italy; 3 IRCCS Santa Lucia Foundation, Via Ardeatina, 306 00179 Rome, Italy; 4 Department of Surgery, University of Verona, Verona, Italy; 5 Department of Psychology, Sapienza University, Rome, Italy; 6 Department of Gnathology, Sapienza University, Rome, Italy; 7 Department of Neuroscience, Biomedicine and Movement Sciences, University of Verona, Verona, Italy; 8 Clinical Medicine - Headache Center, Policlinico Umberto I, Rome, Italy; 9 Pain Therapy Hub, Policlinico Umberto I, Sapienza University, Rome, Italy

**Keywords:** neurorehabilitation patients, primary headaches, cranio-facial pain, non-pharmacological treatment, psychological therapy, manual therapy, invasive treatment, ICCPN

## Abstract

**Background:**

Pain is a very common condition in patient undergoing rehabilitation for neurological disease; however the presence of primary headaches and other cranio-facial pains, particularly when they are actually or apparently independent from the disability for which patient is undergoing rehabilitation, is often neglected. Diagnostic and therapeutic international and national guidelines, as well as tools for the subjective measure of head pain are available and should also be applied in the neurorehabilitation setting. This calls for searching the presence of head pain, independently from the rehabilitation needs, since pain, either episodic or chronic, interferes with patient performance by affecting physical and emotional status. Pain may also interfere with sleep and therefore hamper recovery.

**Methods:**

In our role of task force of the Italian Consensus Conference on Pain in Neurorehabilitation (ICCPN), we have elaborated specific recommendations for diagnosing and treating head pains in patients undergoing rehabilitation for neurological diseases.

**Results and Conclusion:**

In this narrative review, we describe the available literature that has been evaluated in order to define the recommendations and outline the needs of epidemiological studies concerning headache and other cranio-facial pain in neurorehabilitation.

## Introduction

This narrative review is part of the activities of the Italian Consensus Conference on Pain in Neurorehabilitation (ICCPN), an initiative of the Italian Society of Neurorehabilitation – whose members are neurologists, physiatrists, physical therapists and psychologists with a specific and strong background in rehabilitation of neurological disorders - aimed at performing a critical appraisal of the scientific evidence on the role and the consideration of pain in the rehabilitation of neurological diseases (www.sirn.net). The presence of pain, independently from the rehabilitation needs, deserves attention since pain, either episodic or chronic, interferes with patient performance by affecting physical and emotional status. Pain may also interfere with sleep and therefore hamper recovery. Full details of the rationale and the methodology of the ICCPN can be found in a previous publication by Tamburin et al. [1]. The recommendations of our working group on headache and other cranio-facial pains (CFPs) have been recently published in a short report [2]. Here we describe in a narrative form the extensive work of data search and data analysis performed to formulate the recommendations.

In order to comply with the aims and scope of a multidisciplinary approach to primary headaches (PHs) and other CFP in patients undergoing rehabilitation for neurological diseases, the initial team, originally composed by two neurologists with a long-term expertise in headache management and neurorehabilitation (CT and MGB) has been enlarged to include other health professionals: a neurologist with a specific background in pain management (RC), two anaesthesiologists (VS and SB), a psychologist (MD) and three physical therapists MT, VP and RR. A further neurologist (MB) was added with the key role of reference management.

As working tool for defining the search topics, we focused on 6 questions that were formulated by the Board of ICCPN with the primary aim of identifying and summarizing acquired evidence. A secondary important aim was to highlight gaps and critical areas in order to foster future targeted research.

The 6 questions elaborated by the Board of ICCPN are:

1. Do standard methods or criteria exist to evaluate head and cranio-facial pain?
2. What is the specific impact of cephalic and cranio-facial pain in neurorehabilitation?
3. What are the main types of cephalic and cranio-facial pains?
4. Do predictive factors exist to develop cephalic and cranio-facial pain?
5. What is the evidence for using pharmacological and non-pharmacological therapies to treat cephalic and cranio-facial pain?
6. What is the possible impact of treating cephalic and cranio-facial pain on the recovery and neurorehabilitation treatment?

According to ICCPN methodology [1], we searched the main databases for medical literature: Pubmed, Medline, Embase, Scopus and the Cochrane Library. The time period considered was 1984-2017. This time period corresponded to the recommendations of the task force of the ICCP [1] and was ratified by the Authors of the present review based on the results of a preliminary explorative search extended to the previous decade (1974-1983), which did not yield any reliable trials on the use of non-pharmacological treatment in headache and on the consideration that the first evidence-based structured classification of headache disorders was published in 1988. Our review is presented in the form of review is a narrative review, A more capillary search to comply with the criteria of a systematic review was not warranted due to the wide range of topics under evaluation in a single manuscript. For sake of clarity, the keywords used for each question and for each pain disorder are indicated in the corresponding paragraphs. In the initial search, we included all types of peer-reviewed evidence in English language: case-control studies, cohort studies, RCTs, guidelines (GLs), meta-analyses (MAs), systematic reviews (SRs), case reports and expert’s opinion. These two latter types of publication were excluded whenever there was enough evidence from RCTs, GLs, SRs or MAs to answer one or more questions. Reliable GLs were used as very high-level reference. In the other cases, we also considered case reports and experts’ opinions.

## Background and Methods

Among pain syndromes, headaches and other CFPs, either primary or secondary to local or systemic diseases, represent an extremely frequent complaint. The International Headache Society, in 2004, provided a thorough revision of guidelines for proper diagnosis of any head pain by defining, particularly for PHs, clinical characteristics, presentation and recurrence over time (ICHD-II, 2004) [3]. The paucity of standard instrumental pain measure tools enhances the value of individual perception of pain experience, while missing any objective criterion. The ICHD-II, as well as its latest revision (ICHD-IIIbeta, 2013) [4], are subdivided into three main chapters: 1. Primary headaches; 2. Secondary Headaches; 3. Cranial Neuralgias, Central and Primary Facial Pain and Other Headaches. While for PHs, the guidelines propose qualification criteria of pain, for secondary head pains, the international classification does not provide, in most of diagnoses, pain characteristics, whereas it makes mandatory the relationship between organic or systemic conditions possibly responsible for pain. An exception is neuralgic pain, given its typical characteristics. It is intuitive that in the presence of organic or systemic diseases that can cause head pain, the observation tends to answer to the yes/not parameter regarding the relationship between the symptom and the possible cause, therefore moving the attention on the potentially removable or treatable cause of pain rather than on symptom characteristics. A peculiar condition is represented by post-traumatic headache (PTH) [5], more frequently associated to minor head injuries rather than moderate, severe or very severe ones. Up to 90% of subjects with head trauma suffer from PTH, which can persist, at least in a subset of them, for more than 3 months post injury, thus qualifying as chronic PTH, a condition that dramatically affects quality of life and function [6–8]. The mechanism of chronic PTH is currently unknown, and may involve peripheral or central changes, e.g. damage to neck/cranial structures, damage to spinothalamic/thalamocortical pathways, damage/dysfunction of pain inhibition pathways and the frequent association of post-traumatic stress disorder with anxiety, depression and pain catastrophizing [9]. In the case of severe head injuries, other specific factors such as bone fractures, intra- or extra-cerebral haemorrhage, neurosurgical procedures, ventricular dilatation or hydrocephalus are possible sources of pain (ICHD-II, 2004, ICHD-III, 2013) [3, 4]. Minor brain injury *sequelae* do not usually enter the long-term neurorehabilitation processes and are observed in neurology, psychology or as part of litigation procedures. Most frequently, PTHs are described with characteristics that resemble those of PHs, and diagnosed accordingly. This pitfall does not solve the question regarding the specific features of PTH, nor their underlying pathophysiology [5, 10]. Regaining of cognitive functioning in patients with brain injury seems to predict the reappearance of a pre-existing PH, and namely migraine [11].

## Results

In the following paragraphs, we summarize our findings as regards questions n. 1, 3 and 5.

1. Do standard methods or criteria exist to evaluate head and cranio-facial pain?
2. What are the main types of cephalic and cranio-facial pains?
3. What is the evidence for using pharmacological and non-pharmacological therapies to treat cephalic and cranio-facial pain?

In the case of questions n. 2, 4 and 6, we could not find any evidence, and this prevented to draw any evidence-based conclusions or recommendations. Namely, there are no data regarding the impact of PHs and other CFP in patients undergoing rehabilitation for neurological diseases and consequently no data regarding the impact of their treatment in rehabilitation outcome (questions 2 and 6). Concerning question 4, it refers to the neurological diseases undergoing neurorehabilitation and the intrinsic factors of these neurological diseases that may favour the development of primary headaches and other CFP in neurorehabilitation patients. An example of intrinsic factors of other conditions that are capable of interfere with migraine is represented by altered menstrual cycle in young women after severe brain injury following trauma or haemorrhage. Another example is represented by cranio-spinal traumas that may worsen tension type headache with peri-cranial muscle tenderness or contraction. Besides, data should be obtained regarding drugs that are commonly used in neurorehabilitation and their potential impact on PHs or CFPs. This is the case for dantrolene, baclofen and botulin toxin, used for reducing spasticity in several neurological diseases, or for antiepileptic drugs or drugs for the treatment of neuropathic pain in trauma, multiple sclerosis and stroke. Unfortunately, we could find any evidence as regards the predictive factors to cephalic and cranio-facial pain in subjects undergoing rehabilitation for other neurological conditions, with the exception that regaining of cognitive functioning in patients with severe brain injury seems to predict the reappearance of a pre-existing PH, and namely migraine [11].

The association of secondary cephalic pain syndromes with neurological diseases undergoing rehabilitation represented the object of study from other ICCPN groups.

## Q1 – Do standard methods or criteria exist to evaluate head and cranio-facial pain?

The query finds its natural answer in the common use of standard scales to evaluate pain intensity, namely the visual analogue scale (VAS) [12]. Along with this, and limited to migraine, the Migraine Disability Assessment Score (MIDAS) is a useful tool. MIDAS is a very simple questionnaire for patients to quantify the degree of disability due to migraine [13]. The Headache Impact Test (HIT-6) is also recommended for capturing migraine-related disability, but its use is subject to license [14]. The clinical usefulness of MIDAS and HIT-6 is however limited by the lack of questions related to other conditions, such as anxiety and mood disorders, that should be mandatory in the presence of pain. When using the monthly headache diary, usually patients are taught to score their pain on a 0 to 3 scale (0 = no pain, 1 = mild, 2 = moderate, 3 = severe). Along with pain intensity, the diary allows recording of headache frequency and duration (in hours), the presence of premonitory and/or accompanying symptoms to pain, as well as analgesic intake and efficacy (the latter being measurable according to pain relief or cessation after drug intake). This instrument allows to evaluate the efficacy of preventive medications on the long term, through changes in frequency, duration and severity of headache attacks over time. The “diary” therefore emerges not only, or not specifically, as a tool for measuring pain, but more broadly as an instrument to measure the disability related to pain [15–17]. Several other instruments are available from the literature for the evaluation of other dimensions of headache-associated burden, but they do not evaluate specifically pain, therefore we resolved to consider them beyond the scope of this review. An extensive list and associated evaluation is freely accessible via the website of National Institute of Neurological Disorders and Stroke (https://www.commondataelements.ninds.nih.gov/Headache.aspx#tab=Data_Standards).

## Q3 – What are the main types of cephalic and cranio-facial pains?

To answer this specific query, the work group discussed and agreed on the appropriateness to refer to the diagnostic guidelines provided by the International Headache Society (ICHD-II and ICHD-IIIbeta) [3, 4]. These guidelines represent the universal diagnostic tool through which any head or facial pain, either primary or secondary, can be diagnosed following a common language and homogeneous criteria. In the absence of specific data from the literature on the frequency of observation of head pain in the neurorehabilitative setting, it seems reasonable to estimate that the most frequent types of head pain likely to be encountered in the neurorehabilitative setting are tension-type headache and migraine, based on their high prevalence in the general population. PTH can be an acute or chronic *sequela* in post-traumatic patients. The most frequent non-neuropathic pain is Temporomandibular Disorder (TMD), whereas Trigeminal Neuralgia (TN) is the most frequent neuropathic one. Local traumatic injuries (among which tooth extraction are not infrequent) may contribute to local neuropathic pain. Like these conditions, other pain syndromes, such as Burning Mouth Syndrome (BMS) of which little is known [18], require adequate instrumental diagnostic procedures to individuate any possible cause and possible treatment. As suggested by Zakrzewska [19], in a recent review on facial pains, the approach to patients changes according to the first medical referral and management changes as well depending on the current specialist. This apparently useless comment implies that the approach to pain should be on a multidisciplinary basis to ensure adequate management.

## Q5 – What is the evidence for using pharmacological and non-pharmacological therapies to treat cephalic and cranio-facial pain?

Pharmacological and non-pharmacological guidelines for PHs are available by the Italian Society for the Study of Headaches (Società Italiana per lo Studio delle Cefalee, SISC) [20]. These guidelines refer to both pharmacological and non-pharmacological treatment for PH types. We chose to refer to the Italian guidelines since the ICCPN is nationally based. However, they were integrated with a search of literature published after their release, which did not yield further significant information regarding conventional pharmacological therapies. Given the lesser strong evidence for non-pharmacological treatments, a detailed description of the search results is described in the following sections, as summarized in Table 1. Particular emphasis was put on manual therapy and psychological treatment, given their availability in the neurorehabilitative setting, to endorse their use in pain treatment even in those attendant conditions unrelated to the primary disability undergoing rehabilitation.

**Table 1:** Treatments for PHs and other CFP as outlined in the text

Manual therapy
Psychological treatments
Innovative non-pharmacological interventions – Information and communication technologies
Herbal extracts
Acupuncture
Local treatment
Botulinum toxin
Invasive treatment for chronic orofacial pain

### Manual therapy for PHs

An electronic search was done on Medline (Pubmed), Embase and Cochrane Central Register of Controlled Trials and Scopus. The reference lists of potentially relevant trials, meta-analyses and systematic reviews were also searched. The key words and phrases used were migraine, tension-type headache, chronic migraine, chronic daily headache combined with manual therapy, physical therapy or physiotherapy, massage therapy, osteopathic and chiropractic. All RCTs conducted on PHs written in English using manual therapy (MT) were evaluated.

In order to answer the question, the task force, and namely the physical therapists, reviewed all clinical trials and reviews aimed to assess the effects of MT in patients with PHs. Twenty-nine scientific articles were screened for inclusion; 8 of them were excluded because manual treatments were variously combined with nutraceutical treatment, multidisciplinary approach, mental imagery and kinesiotaping; 21 full-articles were reviewed.

For MT, we considered any physical treatment used by physiotherapists, chiropractors, osteopaths, physicians to treat and to manage musculoskeletal disorders and pain. MTs include, but are not limited to, massage, dry needling, soft tissue mobilization, instrument-assisted soft tissue mobilization, rolfing, non-thrust manipulation (mobilization), thrust manipulation (HVLA), myofascial release, strain-counterstrain, muscle energy techniques (MET) spinal manipulation (SM) and osteopathic manipulative treatment (OMT). These manual interventions do not use drugs for the treatment of pain syndromes and/or diseases. Table 2 summarizes levels of evidence and grades of recommendations of MTs for PHs.

**Table 2:** **-** Summary table of levels of evidence [181] and grades of recommendations of manual therapies for primary headaches

Type of manual therapies	Levels of evidence	Grades of recommendations
Chiropractic therapy	1-	C
Osteopathic Manipulative Treatment	1+	B
Myofascial technique	2-	D
Multimodal Approach	2-	D

Legend for Levels of evidence

1++ High quality meta-analyses, systematic reviews of RCTs, or RCTs with a very low risk of bias

1+ Well-conducted meta-analyses, systematic reviews, or RCTs with a low risk of bias

1- Meta-analyses, systematic reviews, or RCTs with a high risk of bias

2++ High quality systematic reviews of case control or cohort or studies; high quality case control or cohort studies with a very low risk of confounding or bias and a high probability that the relationship is causal

2+ Well-conducted case control or cohort studies with a low risk of confounding or bias and a moderate probability that the relationship is causal

2- Case control or cohort studies with a high risk of confounding or bias and a significant risk that the relationship is not causal

3 Non-analytic studies, e.g., case reports, case series

4 Expert opinion

Legend for Grades of recommendations

A: At least one meta-analysis, systematic review, or RCT rated as 1++, and directly applicable to the target population; or a body of evidence consisting principally of studies rated as 1+, directly applicable to the target population, and demonstrating overall consistency of results

B: A body of evidence including studies rated as 2++, directly applicable to the target population, and demonstrating overall consistency of results; or extrapolated evidence from studies rated as1++ or 1 +

C: A body of evidence including studies rated as 2+, directly applicable to the target population and demonstrating overall consistency of results; or extrapolated evidence from studies rated as 2++

D: evidence level 3or4; or extrapolated evidence from studies rated as 2 +

GPP: recommended best practice based on the clinical experience of the guideline development group

#### Chiropractic therapy

A randomized, placebo-controlled, factorial clinical trial of chiropractic and medical therapies in primary care showed that the association of chiropractic treatment (high velocity, low amplitude thrusting manipulation to any dysfunctional joints from occiput to third thoracic vertebrae) with amitriptyline is more effective than each treatment administered individually, in patients with chronic tension-type headache (TTH) [21].

Posadzki and Emst [22] evaluated the effects of SM in patients with migraine: 3 RCTs were published before ICHD-II release, one of them [23] showed that the effect of SM for the treatment of migraine is comparable to placebo, the other two, showed no difference between medication therapy and cervical manipulation [24] and between medication therapy and SM [25] in terms of Headache Index Score and headache duration. In the same year, the Authors [26] published another systematic review to clear up the confusion about the use of SM, concluding that there is no clear evidence of efficacy for SM and emphasized the low quality of the studies included in the revision.

In 2012, the same Authors [27] reported results from 4 RCTs regarding SM for TTHs. Four RCTs, included in the review, suggested that SM was more effective than drug therapy, SM plus placebo, sham plus amitriptyline or sham plus placebo, usual care or no intervention. It is worth mentioning the paper by Chaibi et al. [28] where the Authors propose a protocol design to solve the issue regarding sham intervention to be used as placebo treatment in clinical trials involving SM. A recent three-armed, single-blinded, placebo, randomized controlled trial [29], suggested that the effect of SM in migraine patients is probably due to a placebo effect.

#### Osteopathic manipulative treatment

A RCT by Voigt et al. [30] analysed the effect of osteopathic manipulative treatment (OMT) in female migraine patients and highlighted the statistically significant reduction of pain intensity and pain-related disability in treated vs untreated patients. Cerritelli et al. [31] comparing OMT to drug and sham groups, suggested that OMT is a useful procedure for migraine management. Recently, D’Ippolito et al. reported that after four 45-min OMT sessions patients with high-frequency migraine and comorbid mood disorders showed significant improvement on pain and anxiety disorders [32]. OMT may be preferred over other treatment modalities and may benefit patients with adverse effects to medications or difficulty in complying with pharmacological treatments [33]. In a systematic review, Cerritelli et al. [34] concluded that the evidence supporting the effectiveness of OMT in decreasing pain intensity and frequency, as well as in reducing disability in patients with headache, is still preliminary and methodologically poor.

#### Myofascial release technique

The myofascial release technique proved more effective than the control treatment (simple touch) in patients with episodic and chronic TTH [35]. The efficacy of combination of myofascial muscular and manipulative techniques has been reported for pain-related disability and pericranial tenderness in TTH [36] as well as for pain perception and neck mobility [37]. For both episodic and chronic TTH, joint mobilization, muscle stretching, soft, connective and muscle tissue manipulation were more effective in the short and long period when compared to conventional treatments [38, 39].

#### Multimodal approach

A SR published in 2011 [40] has evaluated the effects of different types of MT (intended as combination of different modalities) in patients with migraine. Seven trials were reviewed, including 6 studies published before ICHD-II [3] release. The results suggest that physiotherapy (massage, muscle relaxation, breathing exercise and thermal bio-feedback) and SM can be equally useful in migraine prophylaxis as propranolol or topiramate, but the methodological quality of the studies included is low, and only four studies showed statistically significant reductions.

A SR [41] assessing the quality of RCTs published from the year 2000 to April 2013 reports that the effectiveness of MT for TTH cannot be completely assessed due to the heterogeneity in study design, outcome measures, and different treatments. Nevertheless, the results suggested that patients with TTH receiving MT (spinal mobilisation, SM, acupressure and OMT) showed better progress than those receiving conventional treatment or placebo. A further SR [42] evaluated the efficacy of MT (massage, mobilization of cervical and thoracic spine, relaxation and stretching) in chronic PHs, showing that MT has an efficacy in the management of chronic TTH that equals preventive medication with tricyclic antidepressants. A SR and MA [43] conducted to evaluate the effectiveness of interventions used by physiotherapists (relaxation, posture correction, exercises, education for muscle tension release, resistance training, orofacial techniques and mobilization) on patient with migraine and TTH showed statistically significant reduction in the intensity, frequency and duration of headache. The Authors concluded suggesting the need of methodological adequate RCTs. Fernández-de-Las-Peñas and Cuadrado [44] state that several types of MT (joint manipulation and/or mobilization, soft tissue interventions, therapeutic exercises and needling therapies) used in a multimodal approach could be effective for headache management.

In summary, studies related to MT do not allow conclusive indications in PHs, although there is some compelling evidence on the efficacy of MT in episodic and chronic TTH. Further studies based upon rigorous methodology are needed. More specifically, careful selection of homogeneous populations, precise evaluation of frequency and duration of treatments (or their association) and comparable methods to assess outcome reliability are needed.

### Manual therapy for other cranio-facial pains

We conducted a search on Medline (Pubmed), Embase and Cochrane Central Register of Controlled Trials and Scopus. The reference lists of potentially relevant trials, meta-analyses and systematic reviews were also searched. The key words and phrases used were orofacial pain, temporomandibular disorders, orthodontic treatment, manual therapy, massage therapy, physical therapy. In the initial search, ninety-eight full papers identified through database searching, twenty-one papers were screened for inclusion and twelve full-articles were reviewed.

Cranio-facial pain (CFP) may be a manifestation of different diseases resulting from periodontal, vascular or sinus bone alterations, cancer or temporomandibular joint disorders (TMJDs) [45]. Cervicogenic headache (CGH), a secondary headache due to neck disorders (Code 11.2.1 in the ICHD-III) [4], is often treated with MT, but pain reduction and frequency reduction do not reach clinically relevant effect [43]. Treatments for TMJ disorders can be non-invasive, invasive and partially invasive [46]. Among the non-invasive non-pharmacological therapies the available choices are occlusal splints, physical therapy, MT and acupuncture. The scientific literature highlights a lack of strong evidence on the effectiveness of orthodontic treatment to treat and prevent TMDs, including occlusal splints that do not significantly reduce the pain [47, 48]. The RCT by von Piekartz and Hall [49] carried out on patients with CGH associated to TMJD, showed that the group that received MT to treat TMJD, in addition to cervical MT, showed significant reduction (that persisted to the 6-month follow-up) in all components of cervical impairment. The same was not observed in the cervical MT-treated group at any point. These observations indicate that manual therapists should look for features of TMJD when examining patients with cervical disorder, particularly if treatment fails when directed only to the latter [50].

Another RCT [51] reports clinical short-term benefit, yet not statistically significant, on pain intensity and joint range with MT and home exercise program. Although Moraes et al. [52] suggest that certain therapeutic exercises can be effective, studies performed to verify the effects of MT used as combination of conservative treatments prevent any attribution of effects to a specific treatment. Overall, systematic reviews show the poor quality of studies and lack of strong evidence for elaborating reliable recommendations. In the absence of evidence-based recommendations, conservative treatment reasonably represents the first choice for any patient. Surgical treatments should be considered only after failure of conservative approaches and should not be proposed in asymptomatic or lightly symptomatic patients [53–56].

### Psychological treatments in PHs

The key words used in the literature search were psychological interventions, cognitive-behavioural therapy, relaxation training, biofeedback, EMDR, mindfulness therapy, hypnosis combined with headache, migraine, tension-type headache and chronic headache. A total of 80 publications were found. The final analysis was conducted on 23 studies.

Psychological treatment is useful to prevent headache progression to chronic form but it should be considered as an integration to pharmacological treatments, rather than an alternative tool. Psychological treatment is also an important part of headache therapy, since it can be used to manage the mood disorders often comorbid in patients with migraine. In fact, many studies suggested that migraine patients are usually comorbid to depression and anxiety [57, 58] which are recognized to influence the prognosis and treatment, being a major risk factor associated with poorer outcome [59, 60].

The comorbidity with major affective disorders is more prevalent in subjects suffering from the chronic type of migraine [61], who frequently report high levels of hopelessness and show a higher suicidal risk [62]. This consideration underscores the usefulness of an appropriate psychological assessment of migraine subjects and the opportunity to associate specific treatment in the subjects with psychiatric comorbidity. Table 3 summarizes levels of evidence and grades of recommendations of psychological treatments for PHs.

**Table 3:** Summary table of levels of evidence [181] and grades of recommendations of psychological treatments for primary headaches

Type of psychological treatments	Levels of evidence	Grades of recommendations
Cognitive-behavioral therapy	1+	A
Acceptance and Commitment Therapy	1+	A
Mindfulness Therapy	4	GPP
Relaxation training	2++	B
Biofeedback	1+	A
Eye Movement Desensitization and Reprocessing	2+	C
Hypnosis	2+	C

Legend Levels of evidence and Legend Grades of recommendations as for Table 2

#### Cognitive-Behavioral Therapy (CBT)

CBT, the most common and successful psychological approach in treating pain, is effective in reducing disability and catastrophic reaction, whereas the effects on pain intensity are negligible [63]. CBT assesses dysfunctional emotions and thoughts and maladaptive behaviours aiming to reduce the emotional and physical suffering associated with pain and to mitigate psychological disorders [64], such as anxiety and mood disorders. Although it is rare for patients to become pain free, CBT helps to reduce pain, by keeping it under control [65]. Patients learn how to manage and therefore to reduce disability and related psychic-emotional worry [66, 67]. Positive outcomes have been detected also in patients with chronic daily headache (with or without medication overuse), when treated with CBT alone or in association with pharmacological treatments [68]. Penzien and coll. [69] found that CBT reduces disability in TTH by 50%, compared to the 33% reduction observed following treatment with amitriptyline. A qualitative study conducted by Morgan et al. [70] on 20 adult migraine patients with at least four headache days per month, reported that CBT was identified by patients as a good ‘tool’ in preventing and managing their attacks and reducing stress. In addition, Gunreben-Stempfle and coll. [71] demonstrated that behavioural management of migraine increases the belief that migraine can be influenced by one’s own behaviour and decreases the belief that migraine is primarily influenced by chance or fate. Systematic reviews [72, 73] stated that the potential effectiveness of CBT in headaches is still unknown, even though it may contribute in the long run to avoid side-effects and costs of medications, as well as other associated health service and personal costs [74].

#### Mindfulness Therapy (Mind-T) and Acceptance and Commitment Therapy (ACT)

During the last two decades, new clinical interventions, i.e. Mindfulness Therapy (Mind-T) and Acceptance and Commitment Therapy (ACT) (third-generation behavioural therapy), have been developed. Mind-T based interventions [75] include meditation and yoga, and focus on the importance of being fully engaged in the present rather than worrying about past or future events. Grant [76] pointed out that meditative practices may positively influence the underlying processes of chronic pain, thus emphasizing their high pain-killer function. A RCT by Cathcart e coll. [77] reported the efficacy of Mindfulness-Based Cognitive Therapy (MBCT) in the treatment of chronic TTH, while Wells e coll. [78], through a pilot study on 19 migraine patients undergoing Mindfulness-Based Stress Reduction (MBSR), reported positive effects on migraine attack duration and related disability, although they failed to demonstrate statistically significant changes in attack frequency and intensity. ACT points to the development of psychological flexibility, the ability to experience the present moment and accept negative thoughts without judgement. Mo’tamedi et coll. [79] showed ACT efficacy in reducing headache disability and its related distress, but not in decreasing the sensory perception of pain in 30 patients suffering from either chronic TTH or migraine without aura. The paucity and limitation of available studies suggest the need to extensively assess ACT before recommending it for the treatment of chronic pain [80], however both ACT and Mind-T show considerable promise for improving outcomes of migraine patients, particularly in reducing headache-related disability and affective distress.

#### Relaxation training (RT)

Relaxation training (RT) is a behavioural technique which helps to break the cycle of stress response favouring physiological and psychological relaxation, which will then facilitate rational and logical thought processes [81]. RT includes progressive muscle relaxation, breathing exercises, autogenic training, guided imagery, meditation and audio-video programs, commonly used as facilitators [82]. RT mitigates sympathetic nervous system excitement and muscle tension to ease body relaxation in order to manage anxiety that can cause headache recurrence [83–85].

Aerobic endurance training (AET) can be also recommended as part of multidisciplinary programs for migraine treatment [86–89], since it has been revealed that it may lead to an increased pain threshold and reduce the migraine attacks frequency, by improving the attentional performance of patients [90]. AET is considered an encouraging alternative for migraine patients, even though high-quality studies are scant [89].

#### Biofeedback (BFB)

Biofeedback (BFB) training has been proven to mitigate migraine, increase RT efficacy and manage pain and stress [91–94]. Patients learn to modulate muscle tension, peripheral body temperature, heart rate, by means of self-control and relaxation techniques, in order to reduce or eliminate headache symptoms [95]. In particular, electromyographic (EMG), thermal, and electrogalvanic BFB interventions are effective in patients involved in multidimensional programs, in addition to CBT, or administered as single treatment [96]. The US Headache Consortium [97] assigned the highest level of evidence to thermal and EMG-BFB in migraine treatment and stress management, and the meta-analysis conducted by Nestoriuc et al. [98] stated that BFB interventions can be effective for the treatment of TTH and migraine. According to Lake [99], behavioural treatments, such as BFB training and relaxation therapy, may have a prophylactic efficacy in migraine.

#### Eye Movement Desensitization and Reprocessing

Eye Movement Desensitization and Reprocessing (EMDR), aiming at reducing the long-lasting effects of distressing memories by developing more adaptive coping mechanisms [100], may be effective in the treatment of chronic pain. An observational clinical trial using a pre-test/post-test design with a 3-month follow-up [101] investigated the efficacy of EMDR in migraine patients by specifically treating traumas related to headaches (e.g., first experienced/remembered headache attack and recalled traumatic events associated to the first headache attack). Patients received an average of eight 50-min sessions on a weekly basis over a 3-month period. At the 3-month follow-up, significant decreases in headache frequency and duration occurred, with less analgesic intake, but no reduction in pain intensity.

#### Hypnosis

Hypnosis may be used on adult patients with migraine and headache [102–104] and it is advised for patients with Persistent Idiopathic Facial Pain [105] or temporomandibular disorders (TMDs) [106, 107].

### Psychological treatments for other CFPs

The key words used in the literature search were psychological interventions, cognitive-behavioural therapy, relaxation training, biofeedback, EMDR, mindfulness therapy, hypnosis combined with facial pain, facial neuralgia, trigeminal neuralgia and temporomandibular disorders.

TMDs are major causes of non-dental pain in the oro-facial region, and may also induce headache [108]. The International Classification of Headache Disorders, 3rd edition (ICHD-3) [4] recognizes headache attributed to TMDs and identifies specific criteria but, since headache and TMDs are very frequent, have multifactorial origins, and have similar or overlapping symptoms, diagnosis is often confused. A key component in the management of patients with TMDs is the behavioural modification of maladaptive habits [109]. A review by Aggarwal and coll. [110] underlined that CBT, either alone or in combination with BFB, can improve outcomes for patients with TMDs in secondary care. However, further research is needed to assess its efficacy in primary care and in management of other chronic orofacial pain conditions [111]. The emphasis is on a medical multidisciplinary model similar to those used for other musculoskeletal disorders [112]. It is also important to tailor treatment to individual patient needs and preferences [113].

Patient education is a significant part of pain therapy, since it can improve the adherence to non-pharmacological therapy by increasing self-efficacy and influencing the locus of control. By improving knowledge of their headache disorder, patients may be more successful in managing their disease, being active in the therapy [114]. It is conceivable that patient education, as part of multidisciplinary treatment, may contribute to improve chronic pain syndromes [114].

### Innovative non-pharmacological interventions – Information and communication technologies (ICT)

Among the more innovative non-pharmacological approaches to headaches, it is noteworthy mentioning the international project coordinated by the National Neurological Institute “C.Mondino” (Pavia, Italy) (funded by the European Commission), which proposes the adoption of a headache diary supported by alerts and alarms for the patients and the doctors (www.comeostas-project.eu) as an adjunct tool for the management of medication overuse headache (MOH). A said above, patients suffering from headache are usually asked to record their attacks. The diary results in a useful tool to monitor pain and drug use/overuse, as well as to obtain an improved control of relapses into overuse [115]. Very little is known about the applicability of electronic diaries in MOH patients, but their implementation seems important also as mean for empowering the patients in the self-management of their condition. Encouraging results have been reported by Sorbi and coll. [116] in a pilot study conducted on 100 patients with chronic migraine, which demonstrated the feasibility and acceptability of an online digital assistance (ODA), intended as an adjuvant to face-to-face or Internet-based cognitive behavioural treatment. ODA application was designed to support home-based training of behavioural attack prevention in chronic migraine, focusing on the identification of attack precursors and the support of preventive health behaviour. According to the Authors, ODA seems to offer a generic tool to combine mobile coaching with diary monitoring, independently of time and space. The effectiveness of these ICT tools on headache improvement remains to be established. Although several qualified studies are published regarding internet-delivered psychological therapies, there is insufficient evidence to draw conclusions regarding this approach [117].

### Herbal extracts

This topic has been subject to review for the implementation of the SISC guidelines [20], already mentioned, to which reference should be made for PHs. More recent studies seem to suggest a possible role of Chinese herbs, but the description of active principle is lacking and the quality of studies is low, especially for characterization of patients and interventions [118, 119]. No scientific evidence was found on other CFP and treatment with herbal extracts.

### Acupuncture

An electronic search was done on Medline (Pubmed), Embase and Cochrane Central Register of Controlled Trials and Scopus. The key words and phrases used were facial pain, facial neuralgia, trigeminal neuralgia, headache, migraine, TTH combined with acupuncture.

Acupuncture treatment seems effective up to 3 months in TTH; efficacy on migraine attacks has been reported but data are weak and as regards migraine prevention data are still controversial. As stated in SISC guidelines [20], the control (sham-acupuncture) utility and efficacy has not been clarified yet. Acupuncture has been used to treat other orofacial pain syndromes. In the controlled study conducted by Simma et al. [120], acupuncture showed an immediate effect higher than the sham treatment. However, the limited number of patients and inadequacy of sham treatment do not allow conclusive results although a systematic review highlights the short-term utility of acupuncture (compared to sham acupuncture) in muscle-originating facial pain [121]. It is extremely difficult to quantify the real effectiveness of active acupuncture treatment. As recently discussed, another major issue is represented by the variability of methodology (needle positioning, treatment duration, session frequency), which contributes to outcome variability [122]. Both reviews by Yang et al. [123] and Linde et al. [124] conclude that *verum* acupuncture may have a higher effect than sham acupuncture over migraine recurrence, but rigorous RCTs are needed. A recent trial [125] concluded that acupuncture may be associated with long-term reduction of migraine recurrence compared to sham acupuncture. However, as stated by Gelfand [126], the placebo effect linked to the *Deqi* sensation (numbness, soreness and distension induced in acupuncture group, and not in sham group) may be responsible for the apparent efficacy as placebo effect. The issue remains, therefore, controversial.

### Local treatment

An electronic search was done on Medline (Pubmed), Embase and Cochrane Central Register of Controlled Trials and Scopus. The keywords and phrases used were topical treatments combined, topical lidocaine, topical ketamine, topical amitriptyline, topical clonidine, topical capsaicin combined with neurofacial pain, localized neuropathic and chronic pain.

The possibility of topical administration of pain killers has been developed nearly 20 years ago. Since then, topical medications have been made available as gel, cream, spray, mouthwash and even lollipop. The effects of capsaicin, lidocaine, gabapentin, clonazepam, amethocaine delivered as topical therapies are well known in orofacial pain syndromes. Local pharmacological therapies are available as topical or trans-dermal preparations [127, 128]. Topical treatment is defined as local application of substances that are absorbed locally and have an effect confined to the site of application. This is also the case of endo-oral application such as lidocaine in dental procedures. Alternatively, trans-dermal absorption provides local absorption and systemic effects (other examples are nitrates or hormonal or nicotine delivery). The drug, locally administered, is intended to stimulate local receptors to reduce local nociceptive “traffic”, thus inhibiting hyperactivity and central sensitization. Mason et al. [129], after reviewing 16 meta-analyses, concluded that the reliability of capsaicin efficacy on neuropathic and muscle-skeletal pain is not guaranteed, even though the substance is more effective than placebo. The evidence from controlled trials on the efficacy and tolerability of topically applied, high-concentration (8%) capsaicin in chronic neuropathic pain in adults is reported by Derry et al. [130] who also report that the additional proportion of subjects who benefit over control is not large, but for those who do obtain high levels of pain relief there are additional improvements in sleep, fatigue, depression and quality of life. However, as Authors highlight, even when efficacy is established, there are unknown risks following repeated application on the long run, especially on epidermal innervation. Conversely, there are insufficient data to draw any conclusions about the efficacy of low-concentration capsaicin cream in the treatment of neuropathic pain [131]. A retrospective study shows that 8% capsaicin patch induces relief of pain up to 12 weeks in patients with severe neuropathic pain. The effect is fast, starting right after placement, it is well tolerated, but it is cost-limited [132]. Cochrane Library reviews evaluated efficacy and tolerability of capsaicin and lidocaine in HIV-post-herpetic neuralgia, peripheral neuropathies and osteoarthritis [133]. Capsaicin, either at low (0,075%) or high (8%) concentration, is able to induce relief in chronic neuropathic pain in adults with limited and transient side effects. Low dose-capsaicin cream shows better efficacy compared to placebo, whereas high dose-capsaicin creams have higher efficacy on pain as well as on pain-related symptoms, such as sleep and life quality. High dose capsaicin patch provides efficacy equal to other systemic therapies. However, the high cost and the permanent disruption of local peripheral sensory fibres should advise to limit its use to those cases unresponsive to other therapies and to avoid repeated local delivery in the absence of severe pain. Topical lidocaine has a low impact on pain and it cannot be recommended as local therapy for neuropathic pain [134, 135]. Local treatment associated with systemic drugs have been suggested for the BMS [136–138]. A review [139] on this issue outlines the efficacy of a combined treatment approach.

### Botulinum toxin type A (BTX-A) for PHs

The key words used in the literature search were botulinum toxin combined with migraine, tension-type headache, chronic migraine and chronic daily migraine. As already reported in the SISC guidelines [20], as regards to the primary episodic headaches, BTX-A has shown good efficacy in several open-label studies while contradictory results have emerged from double-blind-controlled-placebo-studies. The studies carried out have used different protocols, both individualized and standardized, different sites of inoculation and dis-homogeneous groups of patients (especially in terms of attack frequency). A meta-analysis subsequent to SISC guidelines [140] did not confirm the efficacy in episodic PHs, at variance from what initially indicated by open-label studies. Regarding chronic headaches, and as already indicated by the SISC guidelines [20], the data relating to BTX-A controlled-studies in chronic migraine support its effectiveness on multiple indicators of headache, disability and patient quality of life. This effect is comparable to that of drugs of proven efficacy in chronic migraine such as topiramate. The meta-analysis by Jackson et al. [140] confirms the efficacy of BTX-A in chronic daily headache and chronic migraine. The randomized-placebo-controlled Phase III REsearch Evaluating Migraine Prophylaxis Therapy (PREEMPT) studies, extended to 56 weeks of observation, shows the greater effectiveness of BTX-A compared with placebo in chronic migraine, although there is not a direct comparison with other active drugs, and although the placebo effect is quite high [141, 142]. A subsequent analysis of the PREEMPT data [143], aimed at evaluating the effectiveness of BTX-A on subjects with chronic migraine and acute medication overuse, showed that BTX-A is more effective than placebo in reducing headache days, but it does not significantly reduce the consumption of overused symptomatic drugs, except for the triptans. The retrospective study of Lin et al. [144] on the efficacy of BTX-A in refractory chronic migraine has the limitation to have included in the review subjects treated with different protocols and with a follow-up certainly shorter than the PREEMPT study mentioned above, and therefore does not offer further evidence than those already known. As outlined by Silberstein et al. [145], similarly to other preventive drugs, repeated injections (at least two or three) are recommended to evaluate response to the treatment. The confirmation of the efficacy of Onabotulinumtoxin A at the dose of 195 U in Medication Overuse Headache came from the largest post-marketing two years open-label prospective study by Negro et al. [146].

### Botulinum toxin type A (BTX-A) for other CFPs

An electronic search was done on Medline (Pubmed), Embase and Cochrane Central Register of Controlled Trials and Scopus. The key words and phrases used were botulinum toxin combined with trigeminal neuralgia and facial pain.

BTX-A has acquired a further field of application represented by trigeminal neuralgia (TN) refractory to drug therapy for which reference is made to the guidelines of the European Federation of Neurological Societies (EFNS) [147]. The first open-label study, on the efficacy of BTX-A in the treatment of TN was published in 2002 [148]. The Authors report a 50% reduction of pain and frequency of attacks in 8 of 11 patients who were enrolled in the study and treated with 25-75 IU. These results were later confirmed in equally limited numbers of patients: 8 patients, in an open label study [149]; 13 patients, in another open-label study [150]. Zúñiga et al. [151], confirmed in a further open-label study conducted on 12 patients, the immediate disappearance of pain in 10 subjects, and the disappearance of the trigger zone within 2 weeks of treatment with BTX-A, while Bohluli et al. [152], in an open-label study on 15 patients, showed that after BTX-A administration, 7 patients no longer required any pharmacological treatment, 5 patients could successfully manage residual pain with nonsteroidal anti-inflammatory drugs (NSAIDs) and 3 subjects returned to be sensitive to pharmacological therapy with anticonvulsants. The first randomized, placebo-controlled, double-blind study was published in 2011 by Sirois [153]. In this study, the Author introduced a parameter to quantify the effect id BTX-A: the percentage of global pain relief which was considered significant when higher than 50%. The Author concludes that, limited to the number of patients assessed, BTX-A may represent an effective treatment for TN. Those findings were confirmed in a subsequent randomized, placebo-controlled, double-blind study conducted on 42 patients [154]. In particular, the Authors report a significant reduction of pain in the second week after injection with BTX-A and a reduction in frequency already from the first week; the effects were maintained throughout the duration of the study (12 weeks). These data were confirmed by Shehata et al. [155] in a similar experimental design on 20 patients. Cruccu and Truini [156] evaluated the published studies and case reports and rated the results as excellent: the percentage of response in 81 patients treated is 85%, a percentage comparable to that achieved by pharmacological treatment with carbamazepine/oxcarbamazepine, with a mean duration of effectiveness of 105 days. It must be however noted that Verma [157] came to opposite conclusions as he rated the scientific reports available at that time insufficient to define effective BTX-A in the treatment of TN. A similar opinion was also expressed by Hu et al. [158] who identified in the variability of the procedures and in the low number of patients the greatest limitations preventing to recommend BTX-A as a therapeutic option in NT. The advantage of BTX-A, as compared to invasive ablative methods is instead underlined by Guardiani et al. [159] who encourage further randomized controlled trials. Finally, an additional indication to the use of BTX-A is represented by hemifacial spasm. The review by Costa et al. [160] reports the results of large case-control studies that show a rate of benefit between 76 and 100%. Despite the scarcity of good quality data, all available studies suggest that BTX-A is effective and safe for treatment of hemifacial spasm.

### Invasive treatment for chronic orofacial pain

An electronic search was done on Medline (Pubmed), Embase and Cochrane Central Register of Controlled Trials and Scopus. The keywords and phrases used were facial pain, facial neuralgia, trigeminal neuralgia, glossopharyngeal neuralgia, atypical facial pain and neuroablative therapy, microvascular decompression, surgical therapy, invasive therapy or treatments, percutaneous techniques, neurostimulation, neuromodulation, acupuncture, anaesthetic blocks, rehabilitation, physiotherapy, physical therapy. Thirty-one adequate studies were identified (5 meta-analysis, 4 RCTs, 6 prospective non-randomized study, 2 reviews, 2 retrospective studies, 2 case series, 1 case report) and evaluated. Invasive procedures in chronic facial pain are used when drug therapies or other local non-ablative treatments have been unsuccessful, poorly tolerated or not feasible because contraindicated in the specific patient. Literature concerning invasive procedures in chronic orofacial pain are described according to specific diagnosis.

#### Trigeminal neuralgia

According to Gronseth et al., trigeminal neuralgia refractory to pharmacological therapy may be treated with neurosurgical procedures like microvascular decompression (MVD), Gasserian ganglion percutaneous denervation’s or stereotactic radiosurgery (Gamma Knife) [161]. There is very low-quality evidence for long term pain relief of these procedures, while some patients may experience transient or permanent sensory deficits [162]. Regarding MVD, some data suggest better efficacy compared to Gamma Knife or percutaneous techniques although no studies demonstrate a longest pain relief [162, 163]. Pollock et al. reported that MVD has the best cost/benefit ratio in the long term and should be the first choice for patients who can undergo general anaesthesia [164]. Gasserian ganglion percutaneous denervation’s like radiofrequency rhizotomy (RFT), retrogasserian glycerol injection or Gasserian balloon compression are employed in patients without evidence of neurovascular conflict or in case of contraindication to MVD or general anaesthesia but there are a lack of controlled clinical trials comparing these three different techniques [165]. A prospective, non-randomized study shows that retrogasserian glycerol injection and RFT can induce a moderate reduction in pain in refractory trigeminal neuralgia [166]. Finally, Gamma Knife radiosurgery may be considered as first line approach in aged patients, in patients with multiple sclerosis or in case of MVD inefficacy [167].

#### Glossopharyngeal neuralgia

The features of glossopharyngeal neuralgia pain are similar to those of TN. Pharmacological therapies for TN are frequently unsuccessful in glossopharyngeal neuralgia. Invasive procedures are employed but data regarding efficacy are lacking [168].

#### Temporomandibular joint disorder (TMJD)

Although the review by de Souza et al. [53] includes three RCTs, it does not provide conclusive indications about the local drug infiltration in the osteoarthritic TMJD. A prospective randomized study comparing different combinations of arthrocentesis of the temporomandibular joint associated with injection of low or high molecular weight hyaluronic acid or cortisone demonstrates the effectiveness of different treatments without statistical difference between them [169].

#### Facial neuralgia (facial not trigeminal typical pain)

Two selected RCTs evaluating the block of the stellate ganglion [170] and acupuncture in myofascial pain [171] did not provide information on the evolution of chronic facial pain. The study by Salvaggio et al. [172], with a long-term follow-up after stellate ganglion block suggests the usefulness to use it in the early treatment. There are no clinical studies evaluating local anaesthesia in facial neuralgia or facial pain. Published studies include a small sample or case reports.

#### Cluster headache

The greater occipital nerve (GON) block reduces attacks in two thirds of patients with cluster headache [173]. The sub-occipital injection of long half-life steroids was effective in two double-blind controlled studies [174]. Glycerol injection, radiofrequency rhizotomy, microvascular decompression of the Gasserian or pterygopalatine ganglion and the superficial petrosal nerve block or resection are ineffective for the prophylaxis of chronic cluster headache (CCH) even in case of complete trigeminal denervation and may produce complications like secondary trigeminal neuralgia and painful anaesthesia. Gamma Knife radiosurgery showed no medium or long-term effects in front of a high rate of complications and failure of the technique and therefore is not recommended for treatment of intractable CCH [175, 176]. According to Fontaine et al. [177] and Magis et al. [178], occipital nerve stimulation (ONS) may act as a prophylactic treatment in CCH. They suggest that, considering their respective risks, ONS should be proposed before deep brain stimulation in severe refractory CCH patients. The lower posterior hypothalamus stimulation was effective in most patients with non-responsive cluster headache [179, 180]. Sixteen RCTs have been published on ONS or deep brain stimulation in CCH. All these studies show effectiveness of the interventions but with a poor level of evidence. Studies about radiotherapy, sphenopalatine ganglion treatment (electro-stimulation in acute headache, radiofrequency ablation in chronic headache) [181, 182] and occipital injection with steroids have a very low level of evidence (uncontrolled studies including small samples).

## Discussion and conclusions

Before submitting the present review, a further check of salient literature was done and no significant data were found regarding assessment and treatment of PHs and other CFP conditions in patients undergoing rehabilitation for neurological diseases. Treatment of primary or secondary headaches and facial pain is advocated in all conditions and should be pursued also in neurorehabilitative settings in order to alleviate all conditions affecting the patients’ quality of life and to improve their compliance to rehabilitation treatments and outcome. Furthermore, this is a major issue that has legal implications, following the publication of the national law emanated by the Italian Ministry of Health, which requires recording and treatment of any chronic pain in all hospitalized patients, regardless of the disease for which they are under treatment (Gazzetta Ufficiale, 2010) [183]. The lack of observational studies regarding the impact of headaches and CFPs in neurorehabilitation and the impact of their treatment on the rehabilitation outcome does not allow to quantify the burden of these conditions in rehabilitation patients. However, it is desirable that PHs, which are more frequent in young and adult subjects, should be sought and addressed at least in these two populations when they happen to require rehabilitation for other neurological conditions. The same applies for all patients with rehabilitation demanding conditions that are possibly responsible for secondary headaches and CFP. This issue has been addressed by other groups of the ICCPN. It is conceivable that approaching the pain component of the disease (as well as co-existing PHs and other CFPs) in these patients, may improve their psychophysical condition, favour adherence to rehabilitation setting and therefore influence the outcome as well as the quality of life. Furthermore, intrinsic factors of diseases undergoing neurorehabilitation should be carefully evaluated when they represent possible risks for worsening a pre-existing PH or CFP. Along with pain assessment and treatment, sleep disorders should be evaluated in rehabilitation patients, since they may be secondary to nocturnal pain presentation [184]. Diurnal somnolence, lack of concentration, irritability and memory disturbances due to poor quality sleep are additional factors that interfere with rehabilitation programs as well as with pain and produce poor outcome, especially in patients who need cognitive-behavioural rehabilitation following severe acquired brain injury.

To summarize, the final recommendations of the task force on PHs and other CFPs of the ICCPN are:

1. The modality to be used for diagnosing any of the above pain conditions is provided by ICHD with a level of recommendation 1++ and a level of evidence A.
2. For as pharmacological and non-pharmacological treatment of PHs, therapeutic SISC guidelines have been accepted as the recommended reference (1++) with an A level of evidence. Within this reference, the different recommendation levels are provided for any treatment. It is noteworthy that non-pharmacological non-invasive therapies are beneficial: the next step should be to inform patients on the wide range of additional treatments to be combined with the common pharmacological treatments. Non-pharmacological therapies are useful alone or in combination with preventive pharmacological treatment, although scientific evidences and adequate clinical trials are not sufficient for scoring the most of them as highly recommended. Non-drug management should always be considered although the scientific basis is limited.
3. Invasive procedure to treat pain should be regarded as a second order choice, due to the inner risk of any invasive approach. Surgical procedures are not indicated in most patients with cluster headache (grade C recommendation). Patients with chronic intractable headache should be referred to centres with experience in neuro-invasive procedures, as for any other pain syndrome seeking for this kind of approach, and each alternative should be tried before a definitive procedure is performed (good practice point).

As for PHs and other CFP [2], recommendations of the ICCPN have been published in several articles in the October and December 2016 issues of the European Journal of Physical and Rehabilitation Medicine, except for psychological assessment and predictive value of psychological factors in neurorehabilitation patients which is available on April 2016 issue of Frontiers in Psychology Journal [96]. All articles are freely accessible on PubMed and easily found through keyword “ICCPN”. As stated by the Authors, in the editorial that sums up the ICCPN whole work “We hope that the conclusions and recommendations of the ICCPN may both offer practical and useful information on how to deal with pain in patients undergoing rehabilitation for neurological diseases, and represent the basis for future high-quality studies on this topic” [185]. For as PHs and other CFP in neurorehabilitation are concerned, the major issue is the lack of epidemiological data and therefore quality studies are needed to address specific aspects. The availability of multidisciplinary interventions in neurorehabilitation setting should encourage treatment of headaches and other CFP, although independent from the disability that has prompted the need for rehabilitation, to ease the burden and favour the outcome of disabled patients. This would also encourage clinical research in this field to provide better evidences regarding the utility of non-pharmacological, i.e. manual and psychological therapy in PHs and other CFPs. Furthermore and *viceversa*, rehabilitation programs could be proposed as such in chronic headaches and other CFPs refractory to outpatient-based treatments. To conclude, the use of diagnostic (3, 4) and treatment (20) guidelines for headache is highly recommended in the rehabilitative setting for neurological disease other than headaches. In addition to recommended pharmacological options, manual and psychological therapies should be included within a multidisciplinary approach for both PHs and other CFPs, given their widespread availability in neurorehabilitative settings and potential or proved efficacy. Surgical procedures should be proposed in selected conditions only after the failure of adequate conservative approach. Specific, high quality studies are encouraged to increase the evidence and to define appropriate approaches to PHs and other CFPs in the neurorehabilitation setting.

## Abbreviations

ACT: Acceptance and Commitment Therapy
AET: aerobic endurance training
BFB: biofeedback
BMS: Burning Mouth Syndrome
BTX-A: Botulinum toxin type A
CBT: cognitive-behavioral therapy
CCH: chronic cluster headache
CFP: cranio-facial pain
CGH: cervicogenic headache
EFNS: European Federation of Neurological Societies
EMDR: Eye Movement Desensitization and Reprocessing
EMG: electromiographic
GL: guideline
GON: great occipital nerve
ICCPN: Italian Consensus Conference on Pain in Neurorehabilitation
ICHD-II: International Classification of Headache Disorders, second Edition
ICHD-III: International Classification of Headache Disorders, third Edition
ICT: information and communication technologies
IU: International Unit
MA: meta-analysis
MBCT: Mindfulness-Based Cognitive Therapy
MBSR: Mindfulness-Based Stress Reduction
MET: muscle energy techniques
MIDAS: Migraine Disability Assessment Score Questionnaire
Mind-T: Mindfulness Therapy
MOH: medication overuse headache
MT: manual therapy
MVD: microvascular decompression
NSAIDs: nonsteroidal anti-inflammatory drugs
ODA: online digital assistance
OMT: Osteopathic Manipulative Treatment
ONS: occipital nerve stimulation
PH: primary headache
PTH: post-traumatic headache
RCT: Randomized Controlled Trial
RFT: radiofrequency rhizotomy
RT: relaxation training
SISC: Società Italiana per lo Studio delle Cefalee (Italian Society for the Study of Headaches)
SM: spinal manipulations
SR: systematic reviews
TMD: temporo-mandibular disorder
TMJ: temporomandibular joints
TN: trigeminal neuralgia
TTH: tension type headache
VAS: visual analogic scale
